# The impact of perceived English proficiency and competence beliefs on English learning motivation: a structural equation modeling analysis

**DOI:** 10.3389/fpsyg.2026.1927274

**Published:** 2026-09-03

**Authors:** Ming Lyu, Baoqian Yang, Jiabo Li, Yixiao Zhang, Qianqian Wan, Liguo Zhai, Zhuolin Yang

**Affiliations:** 1 School of International Chinese Language Education, Northeast Normal University, Changchun, China; 2 Faculty of Education, Northeast Normal University, Changchun, China; 3 Department of Chinese Language Studies, The Education University of Hong Kong, Hong Kong, Hong Kong SAR, China

**Keywords:** ability mindset, EFL learners, English learning motivation, perceived English proficiency, self-regulated learning

## Abstract

**Introduction:**

In the context of middle school students’ English learning as a foreign language (EFL), perceived English proficiency and ability mindset are two important factors influencing learning motivation. However, the mechanism of their interaction remains lacking in systematic empirical verification.

**Methods:**

This study, using a sample of 2,664 junior high school students from Northeast China, conducted a questionnaire survey using Perceived English Proficiency scales, ability mindset scales, and English learning motivation scales. A structural equation model was constructed to examine the mediating effect of ability mindset between Perceived English Proficiency and learning motivation, and the Bootstrap method was used to test the significance of the indirect effect.

**Results:**

The results showed: (1) Perceived English Proficiency shows a significant positive direct association with English learning motivation; (2) Ability mindset exhibits a partial mediating role as a statistical relationship between the two, and the indirect effect accounts for 8.7% of the total effect.

**Discussion:**

This study provides new empirical evidence for the statistical pathway of ‘Perceived English Proficiency → ability mindset → learning motivation’ in the EFL junior high school population, thereby contributing to the theory of second language motivation from the perspective of positive psychology and also offering potential directions for middle school English teachers to support students’ learning motivation through assessment feedback and growth mindset interventions.

## 1 Introduction

In recent years, the incorporation of individual differences such as psychology, emotion, and cognition into linguistic research has become a major trend. Studying the psychological and cognitive characteristics of learners has risen to become a new direction in foreign language teaching research. Among the individual differences related to second language acquisition, learning motivation is often regarded as a key factor influencing language learning outcomes (Lanvers, 2016). Learning motivation is the internal driving force that guides and promotes students’ learning activities, and motivates them to move toward the designated learning goals and strive. In the English teaching practice in China, scientifically evaluating students’ learning behaviors and effectively stimulating and maintaining their learning motivation have always been one of the important tasks faced by teachers. Existing studies have shown that teachers’ positive evaluations and encouragement can help stimulate students’ learning motivation. However, in traditional classrooms, teachers are usually the only evaluators, and their evaluations have inherent limitations of singleness. Therefore, other evaluation methods, such as Perceived English Proficiency, have increasingly attracted the attention of the academic community and educational practitioners (Tamjid and Birjandi, 2012). It is widely acknowledged that in order to comprehensively and objectively assess students’ true learning performance, diversified assessment methods should be adopted (Harris, 1997). Among various alternative assessment forms, Perceived English Proficiency is of great significance for developing and strengthening students’ independent learning autonomy. Through Perceived English Proficiency, students can more clearly understand their own learning needs, objectively examine their learning performance, and thereby focus their attention on the deep understanding of the learning process, ultimately effectively improving learning efficiency (Larsari and Wildová, 2022). At the same time, the ability mindset, as one of the important individual difference factors for learners, is an individual’s internal belief about the essence of ability. It significantly influences various key aspects such as individual goal setting, learning motivation, and behavioral patterns (Dweck and Yeager, 2019). Discussions around the ability mindset have long been mainly concentrated in the fields of psychology and management, although a few scholars have attempted to explore the relationship between the ability mindset and the investment in mathematics learning, there have been few attempts to systematically introduce it into the field of second language acquisition. Its applicability and explanatory power in the EFL learning context need to be further tested.

Scholars have explored the influencing factors of learning motivation from multiple perspectives, such as self-efficacy, classroom environment, language thinking patterns, and teacher evaluation, all of which have been proven to be closely related to learning motivation. However, the number of studies specifically focusing on the relationship between Perceived English Proficiency and English learning motivation is extremely limited, and the deep mechanism of their interaction has not been fully revealed. More critically, the ability concept, as a cognitive variable closely related to both Perceived English Proficiency and learning motivation, has yet to be studied in the construction of an integrated mediating model among the three. Specifically, there are at least two gaps in existing research: Firstly, although the correlation between Perceived English Proficiency and learning motivation has been preliminarily confirmed in the college student population, whether this relationship is applicable to junior high school students whose academic pressure and cognitive development are at critical stages, lacks direct evidence from large-scale samples; Secondly, existing literature has confirmed the predictive role of the ability concept on learning behavior (Blackwell et al., 2007) and the promoting function of Perceived English Proficiency on autonomous learning (Larsari and Wildová, 2022), but the key cognitive link, that is, how Perceived English Proficiency information is transformed into motivational energy through learners’ belief system about ‘the essence of ability’, has not been directly tested by any empirical research. In other words, the question of ‘how’ and ‘through what path’ Perceived English Proficiency affects learning motivation lacks a clear answer in existing research. Given the aforementioned research gap, this study, using junior high school students as the subjects, constructed a structural equation model with Perceived English Proficiency as the independent variable, learning motivation as the dependent variable, and ability mindset as the mediating variable, aiming to systematically examine the internal relationship among the three, particularly focusing on whether ability mindset plays a mediating role between Perceived English Proficiency and learning motivation and its effect size. Specifically, this study intends to answer the following two questions:

1. What is the relationship between junior high school students’ English Perceived English Proficiency, ability mindset, and learning motivation?
2. Does ability mindset play a mediating role between English Perceived English Proficiency and learning motivation?

This study integrates the self-determination theory and the implicit theory into a unified mediating model in the EFL junior high school student population, providing a new theoretical perspective for understanding the statistical relationships through which individual cognitive factors are associated with learning motivation. At the practical level, the conclusions of this study offer potential directions for middle school English teachers on how to support students’ learning motivation through assessment feedback and belief guidance, thereby bridging the gap between motivation research and classroom practice.

## 2 Literature review

### 2.1 Learning motivation

Learning motivation is the internal driving force behind the occurrence and maintenance of learning behaviors, and it is the intrinsic driving force that promotes and guides students to generate learning needs and desires. In the field of second language acquisition, learning motivation is recognized as one of the key factors influencing the success of learning. In the research spectrum of foreign language learning motivation, the study by Gardner and Lambert (1972) is pioneering. They systematically proposed a classic framework of language learning motivation theory, namely the dichotomous model of integrative motivation and instrumental motivation. Integrative motivation refers to learners’ sincere interest in the target language community and its culture, and their aspiration to integrate into its social life. It is the deep desire of learners to appreciate the target language and its culture and be willing to make continuous efforts to learn and use the target language. Instrumental motivation, on the other hand, refers to learners regarding the target language as a tool to achieve other goals. They hope to obtain certain material benefits or achieve certain realistic goals by mastering the target language, such as passing entrance examinations, obtaining ideal careers, or enhancing social competitiveness.

Based on this, other scholars have expanded and deepened the connotation of motivation from different perspectives. Dörnyei (1994) proposed a triple motivation theory by integrating previous studies, adding achievement motivation (the internal desire of learners to pursue success and self-actualization) in addition to integrative and instrumental motivations. This is manifested as the enjoyment and love for the learning process itself, as well as the positive evaluation and affirmation of one’s own abilities and values. In the 21st century, the research on second language motivation has undergone an important theoretical shift. Dörnyei (2005, 2009) proposed the second language motivation self-system, extending motivation research to the level of learners’ self-awareness. Nguyen (2022) has further consolidated the empirical foundation of this framework across diverse learning contexts. This theoretical shift provides an important background reference for this study to explore the mechanism of motivation generation from the perspective of Perceived English Proficiency and ability beliefs. However, the direct theoretical framework of this study is still based on the Self-Determination Theory and Implicit Theory, as both theories, respectively, explain “how ability mindset drives motivation” and “how ability mindset regulates cognitive assessment.” In foreign language learning, learning motivation has been confirmed by a large number of empirical studies as one of the most effective factors in predicting learning outcomes. For foreign language teachers, only by deeply understanding the types and levels of students’ learning motivation can they adopt matching teaching strategies (Gardner and Lambert, 1972). Stimulating and maintaining students’ English learning motivation has thus become a crucial part of English teaching. Especially for junior high school students, they face the realistic pressure of the high school entrance examination, and the English subject occupies an important position in their academic system. As a variable with the most initiative in the learner’s factors, learning motivation has a significant promoting effect on improving learning performance. Therefore, strengthening the research on junior high school students’ English learning motivation and deeply exploring the individual psychological factors that affect this group’s learning motivation have distinct theoretical significance and practical value.

### 2.2 Ability mindset

The concept of ability is an individual’s internal belief system regarding the essence of ability. It can be divided into the entity theory and the incremental theory. Individuals holding the entity theory tend to believe that human intelligence and talent are fixed entities determined by innate factors; while those holding the incremental theory believe that abilities are malleable and can be developed and enhanced through individual learning and experience accumulation (Dweck et al., 1995). As the core component of the learner’s belief system (Dweck, 1996), the concept of ability significantly influences various dimensions such as an individual’s goal-setting orientation, learning motivation level, and behavioral coping patterns (Dweck and Yeager, 2019). A large number of empirical studies have shown that the concept of ability has a stable and significant predictive effect on an individual’s learning behavior and academic achievement (Blackwell et al., 2007). Meta-analyses (Costa and Faria, 2018; Scherer, 2022) have further confirmed this predictive relationship, while also highlighting the importance of considering contextual and measurement factors.

Students with different views on abilities often exhibit distinct cognitive, emotional and behavioral response patterns during the learning process. They may have different emotional experiences and employ different strategies to deal with difficulties and challenges in learning, thereby having a profound impact on subsequent learning outcomes (Dweck and Molden, 2005). Specifically, students with an entity mindset tend to believe that their abilities are fixed and unchangeable, and thus are more likely to interpret negative feedback as a fundamental threat to their abilities. When facing academic difficulties, they are more likely to lose motivation for learning and experience negative emotions such as boredom and helplessness (Dweck and Yeager, 2019); conversely, students with a growth mindset will pay more attention to the information that is conducive to improving subsequent performance and demonstrate a stronger ability to learn from mistakes and make adjustments (Mangels et al., 2006; Moser et al., 2011). Yeager and Dweck (2020) and Hecht et al. (2021) have has extended these findings by examining the conditions under which growth mindset interventions are most effective, suggesting that contextual factors and developmental stages moderate their impact.

Some studies suggesting smaller overall effects than initially assumed (Macnamara and Burgoyne, 2023), while others have identified significant moderators such as student characteristics and intervention design (Burnette et al., 2023). Thus, it can be seen that the implicit beliefs that learners hold about their own abilities are very likely to have a significant impact on the English Perceived English Proficiency process and learning motivation levels of junior high school students. The deep relationship between the ability mindset and learning motivation requires further empirical investigation.

### 2.3 Perceived English proficiency

In the second language acquisition literature, a distinction should be drawn between self-assessment as a pedagogical process and perceived English proficiency as a static psychological belief. Self-assessment, as a broader concept, refers to an ongoing, formative classroom practice in which learners actively monitor and evaluate their learning progress through activities such as reflection diaries, peer assessment, and rubric-based evaluation. This dynamic process has been widely discussed in the literature for its potential to enhance learner autonomy and self-regulation (Brown, 2004; Butler, 2024; Harris, 1997). In contrast, the present study focuses on a specific psychological component of self-assessment, namely perceived English proficiency, which refers to students’ static perception and evaluation of their current English abilities. This construct captures learners’ beliefs about their own listening, speaking, reading, writing, and overall English proficiency at a given point in time, rather than the ongoing process of self-assessment practice. In the 1970s, the focus of second language research gradually shifted from abstract analysis of the language system to in-depth exploration of the language learning process of learners (Butler, 2024). As an important alternative evaluation form, self-assessment practices began to receive widespread attention from the academic community. The present study adopts a specific operationalization of self-assessment as a static belief construct, namely perceived English proficiency, which captures learners’ evaluations of their own listening, speaking, reading, and writing abilities at the time of the survey.

The educational value of self-assessment practices has been discussed in the literature from multiple perspectives. Firstly, self-assessment activities are regarded as helping learners form an objective self-awareness. Secondly, such practices may enhance students’ learning autonomy and cultivate their ability to monitor and reflect on their own learning progress (Larsari and Wildová, 2022). However, it is important to note that the present study measured perceived English proficiency as a static psychological belief—students’ perceptions of their current abilities—rather than the dynamic process of self-assessment. While the broader literature has discussed the potential benefits of self-assessment practices, our measurement focuses on the belief component, and we therefore cannot directly make claims about the effects of engaging in self-assessment activities.

Brown (2004) pointed out that initiative and independence are the cornerstone of effective learning, and emphasized that developing intrinsic motivation is the most crucial aspect in learning a language or any combination of skills. The active participation of learners can significantly enhance this intrinsic motivation. Similarly, self-assessment practices have been argued to enhance students’ learning motivation and independence by strengthening their clear understanding of educational goals and personal learning needs (Larsari and Wildová, 2022). It should be noted, however, that these studies refer to self-assessment as an ongoing pedagogical practice, whereas our study measures a static belief component. The positive functions discussed by Brown and Liang are potential outcomes of engaging in self-assessment activities, which may not be directly generalizable to the mere perception of one’s abilities.

### 2.4 The relationship between perceived English proficiency, ability mindset, and learning motivation

In recent years, with the vigorous development of positive psychology and educational psychology, researchers have increasingly focused on the intrinsic connections among learners’ emotional factors. For instance, Podsakoff et al. (2003) conducted an earlier study on the relationship between Perceived English Proficiency and learning motivation among college students. Studies have further confirmed the positive association between self-assessment and motivational outcomes in various educational contexts (Mendoza et al., 2023; Namaziandost et al., 2023). She believed that Perceived English Proficiency, as a metacognitive learning strategy, has a close connection with English learning motivation, and empirical research confirmed a significant positive correlation between the two. That is, the stronger and more frequent students’ ability to use Perceived English Proficiency is, the higher their English learning motivation level will be. Moreover, the impact effect of Perceived English Proficiency on integrative motivation and instrumental motivation is not exactly the same. Hosseini and Nimehchisalem (2021) introduced Perceived English Proficiency into the field of primary school English reading teaching, proving that applying the Perceived English Proficiency teaching model in primary school English reading teaching can effectively enhance students’ reading enthusiasm and help them consolidate their dominant position in the knowledge learning process.

In terms of model construction, many scholars have attempted to analyze the complex relationships among various emotional cognitive elements by establishing structural equation models. For instance, Jiang et al. (2019) analyzed the mechanism by which Perceived English Proficiency influences the use of writing self-regulation strategies and writing performance; Yang et al. (2024) explored the relationship between ability mindset and learning engagement, as well as the mediating role of expectations. Additionally, some scholars, based on the Self-Determination Theory (SDT), used structural equation modeling to assess the internalization path of learning motivation, and studied the correlation between student-centered teaching support and the development of students’ self-regulation motivation, autonomy satisfaction, and belongingness satisfaction among college students. They confirmed the mediating role of autonomy satisfaction between student-centered teaching support and competence satisfaction, and emphasized the crucial value of giving students full autonomy in the design of teaching activities by teachers (Larsari and Wildová, 2022). This research complements the study on one of the core elements of student autonomy, Perceived English Proficiency, and jointly reveals the central position of autonomy in the motivation generation chain.

The above research has confirmed from various aspects the predictive effects of Perceived English Proficiency and ability mindset on learning motivation or learning behavior. However, no study has yet examined the intrinsic correlations among these three constructs within the same analytical framework. Specifically, Perceived English Proficiency provides cognitive information about “where I am currently in terms of my level,” while ability mindset determines how learners interpret this information – whether they view the positive Perceived English Proficiency result as a signal that “I am suitable for learning English” or as proof that “my efforts have been effective.” These two different interpretation methods should lead to different motivational consequences. However, this logical chain has not yet been directly tested in existing empirical research. Considering the above research background, ability mindset, as a key cognitive bridge connecting self-awareness and behavioral tendencies, its mediating role in this relationship deserves in-depth exploration.

## 3 Theoretical framework and research hypotheses

The research is based on the dual theoretical foundations of Self-Determination Theory (SDT) and Implicit Theories, and constructs a mediating model to explain the statistical pathway through which Perceived English Proficiency is associated with learning motivation. According to Deci and Ryan (1985)’s Self-Determination Theory, perceived competence is one of the three basic psychological needs that drive the generation of an individual’s intrinsic motivation. Meta-analytic evidence (Howard et al., 2021; Tóth-Király and Morin, 2022) has confirmed the central role of perceived competence in fostering intrinsic motivation across educational settings. When an individual receives positive feedback about their own abilities in a certain activity domain, their need for competence is satisfied, thereby stimulating stronger intrinsic motivation and behavioral engagement. In the EFL learning context, Perceived English Proficiency is an important channel for providing such ability feedback students form an immediate cognition about “how I perform in English” by judging their proficiency in various skills such as listening, speaking, reading, and writing. This positive perceived English proficiency of ability should be theoretically associated with stronger motivation for English learning.

However, this transformation process is not unconditional or mechanical. The way learners interpret the assessment results largely depends on their implicit beliefs about the nature of “ability” – that is, their ability mindset. Dweck’s implicit theory provides a crucial explanatory perspective for this: If learners hold an ability mindset, believing that English ability is determined by innate talent and is fixed and unchangeable, then even if the current Perceived English Proficiency result is relatively positive, they may still maintain a low motivation level due to concerns about not being able to maintain this good state in the future or fearing failure; conversely, if learners hold a growth mindset, believing that English ability can be improved through continuous effort and learning, then the positive Perceived English Proficiency result will be interpreted as “my efforts are effective” or “I have the ability to further improve,” thereby significantly enhancing their subsequent learning motivation and persistence (Dweck and Molden, 2005; Ommundsen et al., 2005).

**Figure 1:**
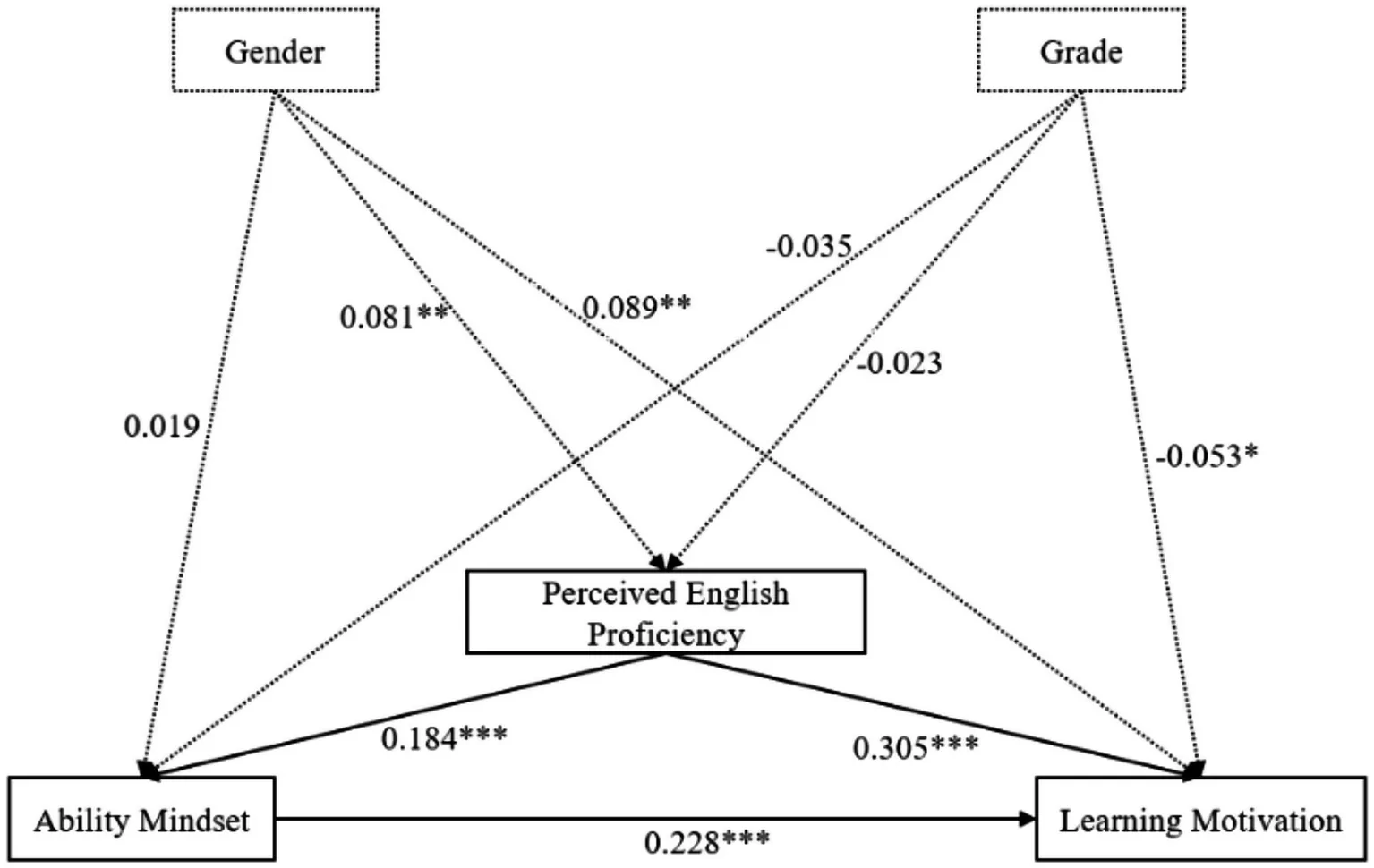
Path diagram of the mediating model. Standardized regression coefficients (β) are presented for each path. All coefficients are standardized. Gender and grade are included as control variables predicting all endogenous variables in the model (perceived English proficiency, ability mindset, and learning motivation). Dashed lines indicate control variable paths. ***p < 0.001, **p < 0.01, *p < 0.05; n.s. = not significant (*p* > 0.05).

From this, it can be seen that the influence of Perceived English Proficiency on learning motivation is not a single direct path, but there is also an important indirect cognitive path: Perceived English Proficiency first activates or strengthens the learner’s belief in the plasticity of abilities, and this belief then promotes the generation and maintenance of learning motivation. In other words, the ability mindset plays the role of an intermediary variable between Perceived English Proficiency and learning motivation. Unlike previous studies that primarily examined bivariate correlations between perceived competence and motivation or between mindset and academic outcomes separately, our model explicitly tests the indirect pathway through which perceived proficiency information is cognitively interpreted via ability beliefs before influencing motivational outcomes, thereby offering empirical evidence for a mediating pathway that has not been widely examined in this integrated framework. Based on the above theoretical deduction, the structural equation model constructed in this study is shown in Figure 1. In this model, English Perceived English Proficiency is the independent variable, English learning motivation is the dependent variable, and the ability mindset is the mediating variable. At the same time, gender and grade are included in the model as control variables predicting all endogenous variables, to control for potential confounding effects of demographic variables.

Accordingly, this study proposes the following two core hypotheses:

*H1*: English Perceived English Proficiency has a significant positive direct predictive effect on learning motivation.

*H2*: The ability Mindset plays a mediating role between English Perceived English Proficiency and learning motivation, that is, Perceived English Proficiency indirectly promotes the enhancement of learning motivation by strengthening the belief in the growth perspective.

## 4 Research methodology

### 4.1 Research subjects and data collection

This study employed the convenient cluster sampling method, selecting 6 middle schools in Northeast China as the sites for the study. The English learners who were studying English as a foreign language (EFL) in these schools were selected as the subjects. The data was collected from June to September 2023. The 6 schools included 2 urban key schools, 2 urban ordinary schools and 2 township schools, located in Changchun City and its surrounding areas of Jilin Province to reflect the differences in educational resources. All schools used the “Go for It!” textbook published by People Education Press, with 5 English classes per week. The tests were conducted in a centralized manner by each class. A total of 3,000 questionnaires were distributed, and after removing the invalid ones, 2,664 valid questionnaires were obtained, with an effective response rate of 88.8%. The age range of the samples was from 12 to 16 years old, with an average age of 13.3 years (SD = 0.714). Among them, there were 1,415 male students, accounting for 53.1%; and 1,249 female students, accounting for 46.9%. The weekly English class hours were 5. Due to the research design, this study did not collect detailed information on students’ extracurricular English learning experiences (such as after-school tutoring, family English environment, etc.). This limitation will be discussed in the research limitations section. All the participants were informed in advance that this survey was anonymous and voluntary, and the questionnaire content did not involve personal privacy. All data will be strictly confidential and will only be used for academic research purposes. During the on-site testing, the participants completed the paper questionnaires in the classroom, and the answering time was approximately 30 min.

Since the data were collected through cluster sampling from multiple school classes, there is a natural nested structure of individual student data at the school and class levels. To account for this clustering effect, we employed cluster-robust standard errors in all path analyses and mediation tests.

### 4.2 Measuring tools

This study collected data through the method of distributing and collecting paper questionnaires on-site. The questionnaire consisted of 19 items, among which Q1 to Q4 were fill-in-the-blank and multiple-choice questions used to collect the basic personal information of the research subjects; Q5 to Q19 were scale contents, covering the following three core measurement dimensions.

#### 4.2.1 English learning motivation scale

This study was based on the Gardner Attitude/Motivation Test Battery (AMTB), and was appropriately simplified and adapted to the actual comprehension ability of junior high school students to measure the English learning motivation level of the participants. The scale consists of 6 items (Q10 to Q15), scored using the Likert 5-point scale, with 5 options provided after each item, ranging from ‘strongly disagree’ (score 1) to ‘strongly agree’ (score 5), allowing respondents to choose based on their own actual situation. The reliability coefficients of each dimension of the scale in this study are: integrative motivation Cronbach’s *α* = 0.81, instrumental motivation Cronbach’s *α* = 0.79, overall reliability Cronbach’s *α* = 0.847, and KMO value = 0.856.

#### 4.2.2 English perceived English proficiency scale

Considering the wide scope of the test and the limited reading comprehension and response time of junior high school students, this study designed a total of 5 items (Q5 to Q9), which, respectively, investigated students’ perceived proficiency in English listening, speaking, reading, writing, and overall ability. This scale was independently developed for this study, with item design referring to the Perceived English Proficiency model of the Common European Framework of Reference for Languages (CEFR) (Council of Europe, 2001), presented in the form of declarative sentences such as “I can.” For example, “I can understand simple instructions in an English class” (listening), “I can have simple daily conversations in English” (speaking), “I can understand the main meaning of English short passages” (reading), “I can write simple English sentences or paragraphs” (writing), “Overall, my English proficiency” (comprehensive). Each item is followed by 5 Likert-type scoring options. Each item provides 5 options, using the 5-point Likert scoring method, ranging from 1 to 5, corresponding to “very low,” “low,” “average,” “high,” and “very high.” This scale has a single-dimensional structure, with excellent internal consistency reliability and ideal structural validity. Before the formal administration of this scale, a pretest was conducted in a non-sample junior high school class in the same region. Based on student feedback, the wording of the items was slightly adjusted to ensure clear and understandable expression.

#### 4.2.3 Ability mindset scale

This study adapted Dweck’s Implicit Theory Scale (Dweck, 2000) into Chinese and designed a total of 4 items (Q16 to Q19) to investigate students’ degree of agreement with descriptions of the nature of ability. The scale consists of 4 items, such as “Whether one can learn English well mainly depends on talent” (ability mindset) and “As long as one is willing to put in effort, anyone’s English can be learned well” (ability mindset). The ability mindset items (Q17, Q19) are scored in reverse, with higher scores indicating a stronger tendency toward the ability mindset. For example: Q16 “As long as one is willing to put in effort, anyone’s English can be learned well” (ability mindset); Q17 “Whether one can learn English well mainly depends on talent” (ability mindset, scored in reverse); Q18 “One’s English proficiency is fixed and difficult to change” (ability mindset, scored in reverse); Q19 “Through effort, I can continuously improve my English proficiency” (ability mindset). The scale is scored using the Likert 5-point scale, ranging from “strongly disagree” (score 1) to “strongly agree” (score 5), with respondents choosing their degree of agreement within the range of 1 to 5. The scale has a single-dimensional structure and shows good reliability and validity, with Cronbach’s *α* = 0.884 and KMO value = 0.825.

### 4.3 Data analysis steps

After data collection is completed, the quality of the data is first reviewed and preprocessed. Regarding missing data, listwise deletion was applied as the proportion of missing data was minimal. The original sample consisted of 2,833 completed questionnaires; after removing cases with incomplete responses (*n* = 169, 5.96%), a final sample of 2,664 valid cases was retained for analysis. Preliminary analysis confirmed that the proportion of missing data was small and unlikely to bias the parameter estimates. The analysis process of this study proceeds as follows: (1) Exploratory Factor Analysis (EFA) – using SPSS 25.0 to conduct dimensionality reduction analysis on all scale items to test the empirical rationality of the three-factor structure; (2) Confirmatory Factor Analysis (CFA) – using AMOS 28.0 to test the fit of the measurement model and the data; (3) Structural Equation Modeling (SEM) path analysis – using maximum likelihood estimation to estimate the parameters; (4) Bootstrap mediation effect test – setting the number of repeated sampling to 5,000 times and calculating the 95% confidence interval. Gender and grade are included as control variables in the model in all analyses.

During the exploratory factor analysis stage, we randomly split the full sample (*N* = 2,664) into two independent halves using SPSS. Sample A (*n* = 1,332, 50% of the data) was used for Exploratory Factor Analysis (EFA), and Sample B (*n* = 1,332, the remaining 50%) was reserved for Confirmatory Factor Analysis (CFA). In Sample A, we employed the Principal Component Analysis (PCA) method to extract factors. The Kaiser–Meyer–Olkin (KMO) measure was 0.851, and Bartlett’s test of sphericity was significant (*χ*^2^ = 8,742.16, *p* < 0.001), confirming the appropriateness of factor analysis. All 15 scale items could be aggregated into three factors with eigenvalues greater than 1. After rotation using the maximum variance method, the factor structure became clear: Factor 1 represented “Perceived English Proficiency” (items Q5-Q9), Factor 2 represented “Learning Motivation” (items Q10–Q15), and Factor 3 represented “Ability Mindset” (items Q16–Q19). The factor loadings of all items were above 0.681, with no cross-loadings exceeding 0.30, and the three factors explaining 68.52% of the total variance (Table 1).

**Table 1:** The results of exploratory factor analysis of the scale (Sample A, *n* = 1,332).

Scale	Question number	EFA 1	EFA 2	EFA 3
Perceived English Proficiency	Q7 Perceived English Proficiency 3	0.862		
	Q6 Perceived English Proficiency 2	0.858		
	Q9 Perceived English Proficiency 5	0.844		
	Q8 Perceived English Proficiency 4	0.831		
	Q5 Perceived English Proficiency 1	0.825		
Learning Motivation	Q14 motivation 5		0.790	
	Q11 motivation 2		0.763	
	Q10 motivation 1		0.725	
	Q13 motivation 4		0.715	
	Q12 motivation 3		0.698	
	Q15 motivation 6		0.681	
Ability Mindset	Q18 Ability Mindset 3			0.866
	Q17 Ability Mindset 2			0.854
	Q19 Ability Mindset 4			0.828
	Q16 Ability Mindset 1			0.816
Total variance percentage		40.82%	16.05%	11.65%
Cumulative variance				68.52%

Extraction Method: Principal Component Analysis. Rotation Method: Varimax with Kaiser Normalization. KMO = 0.851; Bartlett’s Test of Sphericity: *χ*^2^ = 8,742.16, *p* < 0.001.

Based on the EFA results from Sample A, this study further employed AMOS 28.0 software to conduct Confirmatory Factor Analysis (CFA) on the independent Sample B (*n* = 1,332, the remaining 50% of the full sample) to test the fit of the measurement model and the data. Subsequently, a structural equation model (SEM) was constructed using the full sample (*N* = 2,664) and maximum likelihood estimation was used for parameter estimation and path analysis. The evaluation of model fit goodness-of-fit criteria referred to the following standards: GFI, CFI, TLI, IFI, and NFI greater than 0.9 are acceptable, and greater than 0.95 are excellent; RMSEA less than 0.08 is acceptable, and less than 0.05 is excellent. Although the *χ*^2^/df value may be relatively high under conventional standards (<3) when using the full sample, this phenomenon is common in large sample situations (*N* > 2000) because the *χ*^2^ statistic is highly sensitive to the sample size (Bentler and Bonett, 1980; Byrne, 2016). When the sample size exceeds 2000, researchers should rely more on other fit indicators (CFI, TLI, RMSEA, etc.) for comprehensive judgment (Hooper et al., 2008).

Given that the data originate from multiple school classes, individual student data naturally exhibit a nested structure (nested within classes and schools). To account for this clustering effect, cluster-robust standard errors were implemented using Stata’s vce (cluster class id) command in all path analyses and mediation effect tests, thereby controlling for bias arising from non-independence of observations within classes. Specifically, during the Bootstrap sampling process, resampling was conducted at the class level (cluster-bootstrap) to ensure accurate estimation of standard errors.

## 5 Results

### 5.1 Measurement model validation

Before conducting confirmatory factor analysis, the three-factor measurement model was first confirmed through EFA on Sample A (*n* = 1,332). The EFA results (Table 1) showed a clear three-factor structure with all factor loadings above 0.681 and no cross-loadings exceeding 0.30, with the three factors explaining 68.52% of the total variance.

Subsequently, CFA was conducted on the independent Sample B (*n* = 1,332). The CFA results (Table 2) showed that the model fit well: GFI = 0.952, CFI = 0.961, TLI = 0.954, IFI = 0.961, NFI = 0.957, all greater than the excellent standard of 0.95; RMSEA = 0.058, less than the acceptable threshold of 0.08. All the robust indicators performed well, indicating that the three-factor model was stable and reliable, and suitable for subsequent structural model analysis (Bryant and Yarnold, 1995).

**Table 2:** The confirmatory factor analysis results of the three-factor model (Sample B, *n* = 1,332).

Fit indices	GFI	CFI	TLI	IFI	NFI	RMSEA
Tolerance interval	>0.9	>0.9	>0.9	>0.9	>0.9	≤0.08
Statistic	0.952	0.961	0.954	0.961	0.957	0.058

To test the potential impact of common method bias in the full sample (*N* = 2,664), the Harman single-factor test was conducted. A non-rotated principal component analysis was performed on all 15 items, and the result showed that the variance explained by the first factor was 28.67%, which was below the critical standard of 40% (Podsakoff et al., 2003), indicating that the threat of common method bias to the conclusions of this study was within an acceptable range.

To further assess the measurement properties of the three constructs, we examined convergent and discriminant validity. As shown in Table 3, all standardized factor loadings exceeded the recommended threshold of 0.70, and all Average Variance Extracted (AVE) values were above 0.50 (ranging from 0.533 to 0.713), confirming adequate convergent validity (Fornell and Larcker, 1981). Composite reliability (CR) values ranged from 0.872 to 0.926, exceeding the recommended cutoff of 0.70, indicating good internal consistency. For discriminant validity, the square root of AVE for each construct (ranging from 0.730 to 0.844) was greater than the corresponding inter-construct correlations (ranging from 0.269 to 0.499), supporting discriminant validity according to the Fornell–Larcker criterion.

**Table 3:** Convergent validity, composite reliability, and discriminant validity of the measurement model (Sample B, *n* = 1,332).

Construct	Item	Standardized loading	Item reliability (λ^2^)	CR	AVE	√AVE	Correlation with other constructs
Perceived English Proficiency	Q5	0.825	0.681	0.922	0.704	0.839	LM:0.499, AM: 0.269
	Q6	0.858	0.736				
	Q7	0.862	0.743				
	Q8	0.831	0.691				
	Q9	0.844	0.712				
Learning Motivation	Q10	0.725	0.526	0.882	0.555	0.745	PEP:0.499, AM: 0.351
	Q11	0.763	0.582				
	Q12	0.698	0.487				
	Q13	0.715	0.511				
	Q14	0.790	0.624				
	Q15	0.681	0.464				
Ability Mindset	Q16	0.817	0.667	0.905	0.704	0.839	PEP:0.269, LM: 0.351
	Q17	0.855	0.731				
	Q18	0.868	0.753				
	Q19	0.829	0.687				

CR = composite reliability; AVE = average variance extracted; √AVE = square root of AVE. All factor loadings are standardized and significant at *p* < 0.001. PEP = perceived English proficiency; LM = learning motivation; AM = ability mindset.

### 5.2 Descriptive statistics

A descriptive statistical analysis was conducted on the questionnaire responses of 2,664 research subjects (Table 4). The results indicated that the average scores of the learning motivation (*M* = 3.8689, SD = 0.7383), Perceived English Proficiency (*M* = 3.4785, SD = 0.8006), and ability mindset (*M* = 3.4566, SD = 0.9358) dimensions of junior high school students were all higher than the theoretical median, indicating that the sample group generally exhibited a more positive cognitive and emotional tendency in the process of English learning. From the perspective of dispersion, the standard deviation of ability mindset was the largest (0.94), suggesting that there were the most significant individual differences in students’ understanding of the essence of ability; the standard deviation of learning motivation was the smallest (0.74), reflecting that the motivation levels of students were relatively more concentrated. Additionally, the maximum and minimum values of each dimension also indicated that there were certain individual differences within the test group.

**Table 4:** Descriptive statistical analysis.

Variable	*N*	Max	Min	Mean	SD
Learning motivation	2,664	5	1	3.8689	0.7383
Perceived English Proficiency	2,664	5	1	3.4785	0.8006
Ability Mindset	2,664	5	1	3.4566	0.9358

### 5.3 Difference analysis

This study systematically examined the differences of learning motivation, Perceived English Proficiency and ability mindset in terms of two demographic variables: gender and grade.

#### 5.3.1 Gender differences

When analyzing gender differences, an independent sample *T*-test was used. The results are shown in Table 5. In terms of ability mindset, no significant gender differences were found (*p* > 0.05), indicating that male and female students have comparable levels of belief in the essence of ability. However, significant gender differences were found in learning motivation and Perceived English Proficiency (*p* < 0.05). The Perceived English Proficiency of females was significantly more positive than that of males in terms of learning motivation. Specifically, females formed a “double high” pattern in motivation and Perceived English Proficiency, which is in line with the previous research consensus on gender differences in the field of language learning. The standard deviation of Perceived English Proficiency for males was the largest (0.85), suggesting that special attention should be paid to the polarization within the male group in middle school English teaching. Overall, gender has a significant impact on learning motivation and Perceived English Proficiency, so gender should be included as a control variable in the subsequent relationship analysis model.

**Table 5:** Analysis of gender differences.

Variable	Male (*N* = 1,415)	Female (*N* = 1,249)	*p*	*t*
Learning motivation	3.857 ± 0.774	3.883 ± 0.697	0.001	−0.902
Perceived English Proficiency	3.437 ± 0.854	3.525 ± 0.733	0.000	−2.859
Ability Mindset	3.492 ± 0.947	3.417 ± 0.921	0.586	2.054

The *p*-values reported in the table are uncorrected. After Bonferroni correction (α/3 = 0.017), the gender difference in learning motivation remained significant (*p* < 0.001), while the difference in perceived proficiency (*p* = 0.004) also remained significant. The gender difference in ability mindset (*p* = 0.040) did not reach significance after correction.

#### 5.3.2 Grade differences

When analyzing the grade differences, a one-way analysis of variance was employed. The results are shown in Table 6. The mean values of students’ Perceived English Proficiency were very close among different grades, and no statistically significant differences were found (*p* = 0.533), indicating that grade changes did not have a systematic impact on students’ English Perceived English Proficiency levels. There was an extremely significant grade difference in terms of learning motivation (*p* < 0.001). Specifically, the level was the highest in the first grade (*M* = 3.975, SD = 0.638), significantly decreased in the second grade (*M* = 3.768, SD = 0.806), and slightly increased in the third grade (*M* = 3.885, SD = 0.731), showing an overall U-shaped trend. This trajectory is consistent with the general characteristics of junior high school students’ learning psychology in China: newly entering junior high school students have a relatively positive and fresh state, and as the academic difficulty and the pressure of entering senior high school increase, the motivation level declines in the second grade. After entering the third grade, driven by the need for the senior high school entrance examination, the motivation shows a corresponding increase. The grade differences in the perception of ability were also significant (*p* < 0.01). The belief levels of ability for the first-grade students (*M* = 3.514, SD = 0.899) and the third-grade students (*M* = 3.500, SD = 0.939) were similar and relatively high, while the second-grade students were significantly lower than both the first and third-grade students, and were also in a relatively low period. Therefore, in the subsequent path analysis, both gender and grade were treated as control variables.

**Table 6:** Results of one-way ANOVA for grade factors (*N* = 2,664).

Variable	Grade7 (*N* = 820)	Grade 8 (*N* = 988)	Grade 9 (*N* = 856)	*F*	*p*
Learning motivation	3.975 ± 0.638	3.768 ± 0.806	3.885 ± 0.731	18.141	0.000
Perceived English Proficiency	3.489 ± 0.743	3.492 ± 0.836	3.453 ± 0.812	0.629	0.533
Ability Mindset	3.514 ± 0.899	3.370 ± 0.956	3.500 ± 0.939	6.721	0.001

### 5.4 Correlation analysis

Through Pearson’s two-tailed correlation analysis, the correlation relationships among learning motivation, Perceived English Proficiency and ability mindset were examined. Table 7 shows that there are significant positive correlations among the three variables. Specifically, learning motivation and Perceived English Proficiency have a moderately strong positive correlation (*r* = 0.499, *p* < 0.01), learning motivation and ability mindset have a moderately low positive correlation (*r* = 0.351, *p* < 0.01), and Perceived English Proficiency and ability mindset also have a significant positive correlation (*r* = 0.269, *p* < 0.01). The correlation coefficients among all variables have reached the statistical significance level, and the directions are consistent with the theoretical expectations, providing preliminary data support for the subsequent examination of the mediating effect.

**Table 7:** Pearson correlation analysis.

Variable	Learning motivation	Perceived English proficiency	Ability mindset
Learning Motivation	1		
Perceived English Proficiency	0.499**	1	
Ability Mindset	0.351**	0.269**	1

∗∗*p* < 0.01, two-tailed test.

### 5.5 Structural equation modeling and path analysis

This study takes Perceived English Proficiency as the independent variable, learning motivation as the dependent variable, and ability mindset as the mediating variable. At the same time, gender and grade are included in the model as control variables predicting all endogenous variables (perceived English proficiency, ability mindset, and learning motivation). The relevant parameters are estimated using the maximum likelihood estimation method, and a structural equation model is constructed. The model’s various fitting indicators meet the statistical requirements (Table 8): CMIN/DF = 8.112, GFI = 0.958, CFI = 0.963, TLI = 0.957, IFI = 0.963, NFI = 0.958, all above the excellent standard of 0.95; RMSEA = 0.052, below the acceptable threshold of 0.08.

**Table 8:** Model fit test.

Fitindices	CMIN/DF	GFI	CFI	TLI	IFI	NFI	RMSEA
Tolerance Interval	<3	>0.9	>0.9	>0.9	>0.9	>0.9	≤0.08
Statistic	8.112	0.958	0.963	0.957	0.963	0.958	0.052

Regarding the CMIN/DF value, although it is relatively high under the conventional standard (<3), this phenomenon is widespread in large sample situations (*N* > 2000) because the *χ*^2^ statistic is highly sensitive to the sample size (Bentler and Bonett, 1980; Byrne, 2016). When the sample size exceeds 2000, the conventional threshold of CMIN/DF is no longer diagnostic, and researchers should rely more on other fit indicators (CFI, TLI, RMSEA, etc.) for a comprehensive judgment (Hooper et al., 2008). The CFI, TLI, etc. indicators of this study all reach excellent levels, and RMSEA is also within the acceptable range. Comprehensive judgment indicates that the model fits well. The path diagram of the structural equation model is shown in Figure 1.

The model was specified with gender and grade as exogenous control variables predicting all endogenous variables (perceived English proficiency, ability mindset, and learning motivation). The path analysis results (Table 9) show that, after controlling for gender and grade, all direct paths reached significant levels.

**Table 9:** The results of the path relationship test for the structural equation model.

Path relationship	Estimate	S. E.	C. R.	*p*	95% CI
Learning Motivation ← Perceived English Proficiency	0.305	0.015	12.478	<0.001	[0.275, 0.335]
Learning Motivation ← Ability Mindset	0.228	0.015	15.187	<0.001	[0.198, 0.258]
Ability Mindset ← Perceived English Proficiency	0.184	0.015	20.356	<0.001	[0.154, 0.214]
Perceived English Proficiency ← Gender	0.081	0.025	3.241	<0.01	[0.031, 0.131]
Perceived English Proficiency ← Grade	−0.023	0.025	−0.916	0.360	[−0.073, 0.027]
Learning Motivation ← Gender	0.089	0.025	3.562	<0.01	[0.039, 0.139]
Learning Motivation ← Grade	−0.053	0.025	−2.117	<0.05	[−0.103, 0.003]
Ability Mindset ← Gender	0.019	0.025	0.757	0.449	[−0.031, 0.069]
Ability Mindset ← Grade	−0.035	0.025	−1.395	0.163	[−0.085, 0.015]

CI = confidence interval estimated using bias-corrected bootstrap with 5,000 resamples.

Specifically, the path coefficient (standardized) of Perceived English Proficiency on learning motivation was *β* = 0.305 (*p* < 0.001, 95% CI [0.275, 0.335]), indicating a positive statistical association between perceived English proficiency and learning motivation. The path coefficient (standardized) of the ability mindset on learning motivation was *β* = 0.228 (*p* < 0.001, 95% CI [0.198, 0.258]), indicating a positive statistical association between ability mindset and learning motivation. The path coefficient (standardized) of Perceived English Proficiency on the ability mindset was *β* = 0.184 (*p* < 0.001, 95% CI [0.154, 0.214]). The model explained 31.2% of the variance in learning motivation (*R*^2^ = 0.312) and 5.2% of the variance in ability mindset (*R*^2^ = 0.052). This result is consistent with the view that perceived English proficiency plays an important role in the motivation process. A positive perception of language ability is statistically associated with higher levels of learning motivation. Additionally, the significant positive relationship between Perceived English Proficiency and the ability mindset suggests that positive perceived proficiency is associated with a stronger growth mindset belief. Thus, Hypothesis 1 (that Perceived English Proficiency significantly positively predicts learning motivation) has been verified. It should be noted that the path coefficient from perceived proficiency to learning motivation reported in Figure 1 (*β* = 0.305) is the direct effect after controlling for the mediator (ability mindset), which is consistent with the coefficient in Table 9. The total effect (without the mediator) was *β* = 0.482 (Table 10). Both are standardized coefficients. To assess potential multicollinearity among the predictor variables, Variance Inflation Factor (VIF) values were examined. The VIF values for all predictors were as follows: perceived English proficiency (VIF = 1.08), gender (VIF = 1.01), and grade (VIF = 1.01). All VIF values were well below the commonly recommended threshold of 3.3 (Kock, 2015), indicating that multicollinearity was not a concern in this study.

**Table 10:** Mediation effect analysis of ability mindset between perceived English proficiency and learning motivation.

Path	Effect size	95% CI lower	95% CI upper
Total Effect	0.482	0.435	0.529
Direct Effect	0.440	0.392	0.488
Indirect Effect	0.042	0.032	0.052

Bias-corrected Bootstrap with 5,000 resamples. CI = confidence interval.

### 5.6 Mediation effect test

To further verify whether the mediating effect of the ability mindset is significant, this study employed the bias-corrected non-parametric percentile Bootstrap method, setting the number of repeated sampling as 5,000 times. The mediating effect was tested within the 95% confidence interval. The results are shown in Table 10.

The total effect value of Perceived English Proficiency on learning motivation is 0.482 (standardized), with a 95% confidence interval of [0.435, 0.529], and the interval does not include 0, indicating a significant total effect. The direct effect value is 0.440 (standardized), with a 95% confidence interval of [0.392, 0.488], and the interval does not include 0, indicating that the direct effect remains significant after controlling for the mediating variable. The indirect effect value generated by the ability mindset is 0.042 (standardized), with a 95% confidence interval of [0.032, 0.052], and the interval does not include 0, indicating that the indirect effect is significant. The proportion of the mediating effect in the total effect is 8.7%, indicating that the ability mindset plays a partial mediating role between Perceived English Proficiency and learning motivation. Thus, Hypothesis 2 has also been verified. It is necessary to emphasize again that the statistical test of the mediating effect based on cross-sectional data has limited strict causal inference validity, and the results should be regarded as the presentation of the correlation patterns between variables.

## 6 Discussion

### 6.1 The direct relationship between perceived English proficiency and learning motivation

Based on the path analysis of the structural equation model, this study further verified and expanded the previous research results. The results showed that positive Perceived English Proficiency was positively associated with the learning motivation of junior high school English learners. This finding is highly consistent with the conclusion of Lanvers (2016) in the college student group, which reported that the metacognitive strategy of Perceived English Proficiency is positively correlated with students’ English learning motivation. Students’ positive perception of their English ability is statistically related to higher learning motivation, which aligns with the core assertion of Deci and Ryan’s (1985) Self-Determination Theory that perceived competence is an important source of intrinsic motivation.

It is important to emphasize that the following pedagogical suggestions are derived from theoretical logic and the broader literature on self-assessment, rather than directly from our measurement of perceived English proficiency as a static belief. The observed statistical associations between perceived proficiency and motivation suggest potential avenues for practice, but these suggestions require validation through future intervention research. Based on the above findings, it is self-evident that cultivating middle school students’ Perceived English Proficiency ability is of great significance for their English learning. Specifically, positive self-evaluation can help students enhance their self-efficacy, and this confidence may, in turn, support students’ engagement in English learning, potentially encouraging more active participation in English classroom interactions and post-class language practice, which may contribute to the maintenance and development of learning motivation. Therefore, in the practice of middle school English teaching, teachers should abandon the habitual thinking of “only evaluating by scores,” and truly pay attention to the development of learners’ Perceived English Proficiency ability. Through systematic training, teachers could consider developing students’ awareness of Perceived English Proficiency, helping them identify their own strengths and weaknesses in various language skills, and supporting them in establishing a scientific self-evaluation framework from multiple cognitive dimensions, which may contribute to sustaining learning motivation.

### 6.2 The mediating role of ability mindset

The core finding of the research is that the mediating effect of the ability mindset between Perceived English Proficiency and learning motivation has been empirically supported. The correlation analysis indicates that there are significant positive correlations among the Perceived English Proficiency, ability mindset, and learning motivation of junior high school English learners. This provides the necessary prerequisite conditions for the mediation test. On this basis, the structural equation model and Bootstrap mediation test further confirmed the mediating path of the ability mindset-Perceived English Proficiency is not only directly associated with learning motivation, but also exhibits an indirect association through its relationship with students’ growth mindset beliefs. This study integrates Self-Determination Theory and Implicit Theory into a unified mediating model, and empirically examines the cognitive transformation function of ability mindset between perceived proficiency and learning motivation. Unlike previous studies that only focused on bivariate correlations, our findings reveal that the influence of perceived proficiency on motivation is not a straightforward, complete transformation. Instead, it operates partially through altering learners’ beliefs about the nature of ability (i.e., growth vs. fixed mindset). This mechanism suggests that how learners interpret their own ability information is a critical cognitive link determining whether such information can effectively trigger motivation, thereby providing a new theoretical anchor for second language motivation research.

In addition to this theoretical integration, the present results also empirically validate Dweck’s (1996) implicit theory of intelligence in the context of language learning, and are consistent with the findings of Ommundsen et al. (2005). Research has suggested that students with a growth mindset tend to hold more positive expectations for the development of their English proficiency, and tend to believe that English proficiency is improvable through effort and effective strategies. This belief in the malleability of abilities has been found to be associated with more active participation in academic activities and greater investment of time and effort (Ommundsen et al., 2005; Dweck, 2006). In contrast, studies have shown that students with an entity mindset tend to focus more on avoiding negative self-evaluations (Dweck et al., 1995), and may adopt self-impeding strategies (Covington, 2000), such as setting lower academic goals and reducing their willingness to engage in learning, in order to protect their self-worth.

For junior high school students who are in a critical period of academic development, formally incorporating the metacognitive belief of “whether English proficiency can be changed” into the analysis framework of motivation system holds special theoretical significance and practical value. During the process of providing assessment feedback, teachers should consciously incorporate growth-oriented evaluation language to help students gradually establish the internal belief that “ability can be changed through effort,” thereby effectively avoiding the negative psychological consequences that may be brought about by fixed mindsets such as the ‘language talent theory’.”

One notable phenomenon is that the mediating effect of ability mindset in this study accounted for only 8.7%, which was much smaller than the direct effect (*β* = 0.440). This indicates that the promoting effect of perceived ability on learning motivation mainly occurs directly by meeting the basic psychological needs of learners (i.e., the competence need in the self-determination theory), while the indirect path through ability mindset is statistically significant but contributes relatively less to the total association. One possible explanation is that for junior high school students, the immediate evaluation of their own ability level itself has a strong motivational effect – when they think their English is “good enough,” they will naturally have higher learning enthusiasm. Regarding the deep belief of “whether ability can be changed,” its influence may be more fully observed over longer timeframes or in intervention contexts. This finding is consistent with the idea that teachers could first help students recognize their own progress and ability, and then foster a growth mindset orientation on this basis, so as to achieve twice the result with half the effort.

### 6.3 Discussion on gender and grade differences

The differential analysis of this study reveals some patterns worthy of in-depth exploration. In terms of gender, girls consistently outperform boys in Perceived English Proficiency and learning motivation, which is in line with the consensus of numerous studies in the field of gender differences in language learning. From the perspective of cognitive processing methods, a large number of behavioral and neuroscientific studies have shown that women tend to activate a broader brain network in language tasks for information processing. This more distributed processing mode may provide certain advantages for women in language comprehension and production (Jaeger et al., 1998; Kansaku and Kitazawa, 2001). In terms of strategy usage, Oxford and Crookall (1989) pointed out that girls use various language learning strategies more frequently during language learning, including metacognitive strategies and social–emotional strategies, etc. In terms of speech skill development, girls start speaking earlier than boys and acquire vocabulary and grammar at a younger age, and women’s stronger listening comprehension ability in their native language can be transferred to second language learning – even when the reading comprehension level of the participants’ native language is strictly controlled, women still perform better than men in second language reading comprehension (Payne and Lynn, 2011). Moreover, recent analyses based on large-scale educational quality monitoring data further confirm that girls show significant and sustained advantages in language-related academic tasks, and this differential pattern has a high consistency across different cultural backgrounds and educational systems (Reilly et al., 2019). These studies provide evidence from different levels for the superior performance of girls in language abilities, and thus girls’ Perceived English Proficiency of their English proficiency is also higher than that of boys. In terms of learning motivation, girls have stronger motivation for foreign language learning than boys, which is specifically manifested in girls setting higher learning goals, studying more diligently, being more eager to achieve learning goals, and having a more positive attitude toward language learning (Jiang et al., 2019).

It is worth noting that there is no significant gender difference in the ability mindset, indicating that junior high school students of both genders tend to have similar basic beliefs regarding the plasticity of ability. This result is consistent with the macro-level observation on the trend of gender education equality in China by Hosseini and Nimehchisalem (2021), but the individual-level data of this study cannot directly test this macro social mechanism. Future research can further explore the potential influence of family structure on ability beliefs. However, the standard deviation of Perceived English Proficiency for boys is the largest, suggesting that in middle school English teaching, special attention should be paid to the polarization within the male group, and timely intervention should be given to boys with low Perceived English Proficiency.

In terms of grade differences, both learning motivation and ability mindset show significant grade effects, and the trends follow a U-shaped curve, first declining and then rising. Specifically, the scores of students in the first grade of junior high school are the highest. The scores of students in the second grade significantly decrease and reach a bottom, while those in the third grade show a slight recovery. This trend is highly consistent with the educational ecosystem of junior high school in China: Freshmen entering the campus bring a sense of novelty and anticipation for middle school life, and their learning attitude is relatively positive. In the second grade, they face a significant increase in academic difficulty and a period of intense psychological development fluctuations, resulting in a temporary low point in the motivation and belief system. After entering the third grade, the realistic pressure of the middle school entrance examination transforms to some extent into an external driving force, prompting a rebound and recovery in students’ learning motivation and belief. This finding has direct warning significance for teaching practice – the second grade should be regarded as a key window period for motivation intervention and belief guidance. Schools and teachers need to provide students with more psychological support and strategy guidance during this period.

### 6.4 Theoretical contributions and practical implications

From a theoretical perspective, this study integrates the Self-Determination Theory and the Implicit Theory into a unified mediating model, and identifies a statistical pathway through which Perceived English Proficiency is associated with learning motivation. ‘Perceived English Proficiency → ability mindset → learning motivation’. This integrative perspective, which incorporates both perceived proficiency and ability beliefs within a single analytical framework, offers empirical evidence that contributes to the theoretical content of second language motivation research from the perspective of positive psychology. The study found that ability mindset exhibits a mediating role in the relationship between perceived English proficiency and learning motivation, providing a new theoretical anchor for understanding the internal mechanism of how individual cognitive factors affect learning motivation. At the same time, this study responds to Dörnyei’s (2009) theoretical call for incorporating learners’ self-system into motivation research, providing empirical supplementation from the perspective of ability mindset for the second language motivation self-system.

It should be noted that the operationalization of perceived English proficiency in this study focuses on students’ static perception and evaluation of their own language abilities, rather than the process-based self-assessment activities as teaching strategies in the classroom. This is an important conceptual distinction: our measurement captures a psychological belief at a single time point, whereas classroom self-assessment involves ongoing, formative practices such as reflection diaries and rubric-based evaluation. Therefore, the derivation of the following teaching suggestions is based on theoretical logic and the broader literature on self-assessment, rather than direct empirical evidence from our measurement, and their effectiveness awaits further verification through future intervention studies. From a practical perspective, the findings of this study provide clear operational implications for middle school English teaching. Firstly, teachers should attach importance to and systematically cultivate students’ Perceived English Proficiency abilities, helping them establish a scientific and objective self-evaluation system. Specific strategies include regularly guiding students to complete English learning reflection diaries, conducting Perceived English Proficiency training based on rubrics, organizing diversified evaluation activities that combine peer assessment and Perceived English Proficiency, etc. Secondly, in the process of assessment feedback, teachers should consciously integrate a growth-oriented discourse system, for example, emphasizing “progress” rather than “class ranking” when grading homework or making classroom comments, and redefining “mistakes” as “opportunities for learning” rather than “signs of failure.” Through continuous growth-oriented feedback, help students establish a positive belief at the cognitive level that “English ability is dynamically developing and can be improved through effort,” thereby activating the growth perspective at the source of Perceived English Proficiency and effectively driving the continuous generation and positive cycle of learning motivation through this intermediary path. Finally, the findings of this study regarding grade differences suggest that the second grade is a key window period for motivation intervention. Schools and teachers should carry out targeted growth mindset intervention and Perceived English Proficiency training at this stage to prevent the excessive decline of the motivation and belief system.

## 7 Conclusion

This study was based on the empirical questionnaire data of 2,664 junior high school students. A structural equation model was constructed and tested, with English Perceived English Proficiency as the independent variable, learning motivation as the dependent variable, and ability mindset as the mediating variable. It deeply explored the deep cognitive mechanism and the action path through which Perceived English Proficiency affects learning motivation. The main research conclusions are as follows:

First, perceived English proficiency shows a significant positive direct association with learning motivation. Students’ positive perception of their own English proficiency is statistically associated with higher learning motivation, and this association remains after controlling for gender and grade variables.

Secondly, ability mindset exhibits a partial mediating role in the relationship between perceived English proficiency and learning motivation. The mediation analysis shows that perceived English proficiency is not only directly associated with learning motivation, but also indirectly associated through its relationship with students’ growth mindset beliefs, with the mediation effect accounting for 8.7% of the total effect. Specifically, this study verified two paths of action: the first is the direct path of “English Perceived English Proficiency → learning motivation”; the second is the indirect cognitive path of “English Perceived English Proficiency → ability mindset → learning motivation.” However, due to the cross-sectional research design, the above mediation paths should be understood as statistical relationships based on theoretical presuppositions rather than the final confirmation of the causal mechanism.

This study identified the comprehensive influence mechanism of two types of individual psychological factors, namely Perceived English Proficiency and ability mindset, on learning motivation. It demonstrated the statistical pathway through which perceived English proficiency is associated with both growth mindset beliefs and learning motivation. This provides new empirical evidence for the study of EFL motivation from the perspective of positive psychology. At the same time, the findings of this study also pointed out the direction for middle school English teaching practice: Teachers need to promptly pay attention to students’ psychological cognitive states, and help students cultivate a scientific sense of Perceived English Proficiency in a personalized manner, shape a positive and malleable belief in ability, leverage assessment feedback and belief guidance to support learning motivation, and thereby effectively improve students’ language learning outcomes.

## 8 Research limitations and future directions

Although this study has made certain progress in theoretical construction and empirical testing, there are still several limitations that need to be addressed and improved upon in future research.

First, issues related to sample representativeness and data nesting. The participants in this study all came from junior high schools in Northeast China. The English teaching resources, teacher quality, and educational policies in these schools may have specific regional characteristics. The external validity of the research conclusions needs to be further verified in samples from other regions (such as the eastern coast, central and western regions with different levels of educational development). Future research could consider adopting a large-scale sampling design with multiple regions and across provinces to enhance the generalizability and applicability of the research conclusions. Additionally, since the data is derived from cluster sampling of multiple school classes, there is a natural nesting structure at the school and class levels, which may violate the assumption of observational independence. In this study, we addressed this issue by employing cluster-robust standard errors (implemented via Stata’s vce (cluster class id) command) in all path analyses and mediation tests. However, future research could further validate the findings using multilevel structural equation modeling (MSEM) to more comprehensively account for the nested data structure and to partition variance at the school and class levels.

Second, limitations of cross-sectional design and causal inference. In terms of the research design, a cross-sectional survey was adopted, and all variables were collected at the same time point. Therefore, it is impossible to strictly infer the causal relationship between variables. Although this study constructed an intermediary model based on solid theoretical reasoning and observed significant indirect effects at the statistical level, there may be more complex dynamic relationships among perceived English proficiency, ability mindset, and learning motivation, and even the possibility of reverse effects, which our cross-sectional design cannot address. Future research can adopt longitudinal tracking designs or experimental intervention designs to examine the changing trajectories of variables over time and their mutual predictive relationships, in order to more rigorously confirm the causal direction.

Thirdly, common method bias and lack of objective criteria. All the variables in this study were obtained through students’ self-reports. Although the confirmatory factor analysis results indicated that the measurement model was well-fitted, and the Harman single-factor test did not show a situation where a single factor dominated, the self-report data may still be affected by social approval responses, recall biases, and common method variance. Moreover, this study did not include students’ actual English academic performance as an external criterion, so it is impossible to directly answer whether the improvement in Perceived English Proficiency and motivation ultimately leads to observable improvements in learning outcomes. Future research can consider introducing multiple sources of data, such as incorporating teachers’ observation evaluations, peer assessment results, or students standardized English test scores as supplementary criteria, to more comprehensively and objectively assess students’ actual English proficiency and learning performance, and reduce the interference of single-method bias on the research conclusions. At the same time, combining self-report data with objective academic indicators can also help test whether the cognitive path revealed by this study ultimately has a detectable impact on the actual effectiveness of language learning.

Fourth, construct operationalization and measurement transparency. The perceived English proficiency scale in this study focuses on students’ static perception of their own English listening, speaking, reading, writing, and overall proficiency. This measurement captures a psychological belief at a single time point, rather than the dynamic, ongoing process of self-assessment practice in classroom settings. The latter involves formative activities such as reflection diaries, peer assessment, and rubric-based evaluation, which were not measured in this study. This boundary condition should be taken into account when translating the research conclusions into teaching practice. Future research could develop process-oriented instruments that capture students’ engagement in self-assessment activities over time, and examine whether the statistical relationships observed in this study hold under different operationalization methods or in intervention contexts.

Fifth, expansion of mediating variables and motivation subtypes. This study only examined the single mediating variable of ability mindset. Although this variable has a clear positioning in theoretical derivation, the paths by which Perceived English Proficiency affects learning motivation are clearly not limited to one. Future research can incorporate variables such as self-efficacy, academic emotions (such as pleasure and anxiety), and achievement goal orientation into multiple mediation models or chain mediation models to more comprehensively examine the multiple pathways through which perceived English proficiency is associated with learning motivation. At the same time, this study did not make detailed subtype distinctions for English learning motivation but treated it as a whole construct. However, contemporary motivation theory (Dörnyei, 2005, 2009) indicates that learning motivation contains different types of dimensions. Future research can further explore the differential impacts of Perceived English Proficiency and ability mindset on different motivation subtypes to enrich our understanding of the internal structure of motivation.

Sixth, the lack of information on extracurricular learning experiences. This study did not collect detailed information on students’ extracurricular English learning experiences (such as after-school tutoring, family English environment, extracurricular reading volume, etc.). These factors may simultaneously affect students’ Perceived English Proficiency, perception of ability, and learning motivation, and their potential confounding effects were not controlled in this study. Future research should include extracurricular learning experiences in the survey scope or treat them as covariates in the analysis.

In conclusion, despite the above limitations, this study has empirically tested the mediating role of ability mindset between perceived English proficiency and learning motivation at the theoretical level, and provided operational evidence support for assessment reform and motivation intervention in middle school English teaching at the practical level. We look forward to future research being able to continue to deepen the exploration and verification of this cognitive path in a wider sample and more refined research design.

## Data Availability

The raw data supporting the conclusions of this article will be made available by the authors, without undue reservation.
